# Venetoclax treatment for chronic lymphocytic leukemia/small lymphocytic leukemia in Japan: post-marketing surveillance

**DOI:** 10.1007/s12185-024-03832-x

**Published:** 2024-08-21

**Authors:** Tomoki Ito, Tomohiko Kamimura, Toru Kiguchi, Koji Kato, Risa Takenaka, Mariko Kobayashi, Ayumi Ito, Mizu Sakai, Koji Izutsu

**Affiliations:** 1 Department of Hematology, Kansai Medical University Hospital, 2-3-1 Shinmachi, Hirakata City, Osaka Japan; 2 Department of Hematology, Harasanshin Hospital, Fukuoka, Japan; 3 Department of Diabetes, Endocrinology and Hematology, Dokkyo Medical University Saitama Medical Center, Saitama, Japan; 4 Department of Hematology, Oncology and Cardiovascular Medicine, Kyushu University Hospital, Fukuoka, Japan; 5 AbbVie GK, Tokyo, Japan; 6 Department of Hematology, National Cancer Center Hospital, Tokyo, Japan

**Keywords:** Chronic lymphocytic leukemia, Japan, Post-marketing surveillance, Venetoclax

## Abstract

Venetoclax was approved for relapsed/refractory chronic lymphocytic leukemia (R/R CLL) and small lymphocytic leukemia (SLL) in Japan in September 2019; however, clinical data in Japanese patients are limited. This all-case post-marketing surveillance assessed efficacy and safety in Japanese patients with R/R CLL/SLL who started venetoclax treatment between November 2019 and August 2020. Overall, the safety and efficacy analysis sets included 129 and 114 patients, respectively. The overall response rate (ORR) was 57.0%; ORRs were higher in patients with versus without concomitant rituximab (65.4% vs. 54.7%), and in patients with 1 versus ≥ 2 prior lines of therapies (72.5% vs. 44.4%). Adverse events (AEs) were reported in 66.7% of patients (86/129); the most common AEs were neutrophil count decreased (22.5%), white blood cell count decreased (7.8%), and tumor lysis syndrome (TLS; 6.2%). AEs of special interest (TLS, myelosuppression, and infection) were manageable in clinical practice in Japan. Venetoclax is efficacious and safe for R/R CLL/SLL patients in the real-world setting in Japan. *ClinicalTrials.gov ID:* NCT04198415.

**Supplementary Information:**

The online version contains supplementary material available at 10.1007/s12185-024-03832-x.

## Introduction

The treatment landscape of chronic lymphocytic leukemia (CLL) has evolved in recent years with a greater understanding of its pathogenesis, leading to the development and utilization of targeted therapies as the preferred treatment option [1].

Aberrant expression of B-cell lymphoma 2 (BCL-2), an anti-apoptotic protein, is common in CLL [2, 3]. Overexpression of BCL-2 protects leukemia cells from apoptosis by inhibiting the activation of BCL-2-associated X (BAX) and BCL-2 antagonist killer (BAK), which act as key cell death effector proteins [2, 3]. Given its central role in the apoptotic pathway, BCL-2 is a rational therapeutic target in lymphoid cancers, including CLL [2, 3].

Venetoclax is an oral, small-molecule BCL-2 inhibitor that was approved for the treatment of relapsed/refractory (R/R) CLL (including small lymphocytic lymphoma [SLL]) in combination with rituximab in Japan in September 2019 [4, 5]. This approval followed the global phase 3 MURANO study (NCT02005471), in which non-Japanese patients with R/R CLL treated with venetoclax plus rituximab had significantly longer progression-free survival than patients treated with bendamustine plus rituximab [6]. Venetoclax as monotherapy (*n* = 6) and in combination with rituximab (*n* = 6) was also demonstrated to have efficacy and to be well tolerated in a phase 1/2 study (NCT02265731; M13-834) in Japanese CLL/SLL patients [7].

This all-case post-marketing surveillance (PMS) was conducted to report large scale, real-world use of venetoclax in R/R CLL/SLL patients in Japan.

## Materials and methods

### Study design, patients, and conduct

This was a multicenter, prospective, non-comparative, observational, centrally registered all-case PMS in R/R CLL/SLL patients who started venetoclax treatment between November 22, 2019 and August 31, 2020 in Japan (NCT04198415).

Enrolled patients were observed from the first administration of venetoclax until 32 weeks after the conclusion of the dose ramp-up phase. If treatment was discontinued before the end of the observation period, adverse event (AE) occurrences were investigated from the last day of venetoclax administration until 30 days later. Anonymized patient data were collected through electronic case report forms (CRFs) using an electronic data capture (EDC) system.

The study was conducted in accordance with the Japanese Good Post-marketing Study Practice regulations, and the protocol was approved by the Japanese Pharmaceutical and Medical Devices Agency.

### Assessments

Baseline data on patient characteristics and demographics, concomitant medications, venetoclax treatment details, efficacy, and safety were collected during the observation period. For venetoclax treatment details, physicians assessed whether ramp-up phase was completed.

Efficacy assessments included overall response rate (ORR) and the time to best response. Responses were assessed by physicians using the 2018 International Workshop Group on CLL (iwCLL) response criteria [8], and were classified as complete response (CR), complete response with incomplete bone marrow recovery (CRi), partial response (PR), nodular partial response (nPR), stable disease (SD), or progressive disease (PD).

Safety assessment included the incidences of AEs, which were classified using the preferred terms (PTs) of the Medical Dictionary for Regulatory Activities/Japanese (MedDRA/J) version 25.0. The severity of AEs were graded according to the Common Terminology Criteria for Adverse Events (CTCAE), version 5.0. Tumor lysis syndrome (TLS), myelosuppression, and infections were assessed as AEs of special interest.

### Statistical analysis

A sample size of 100 patients was planned for this study based on the incidence of TLS (3.1%) in the global phase 3 MURANO study [6]. Accordingly, approximately 100 patients are required to detect at least one TLS with ≥ 95% probability.

Descriptive statistics were used to summarize data, with medians and ranges (minimum, maximum) used for continuous variables, and number and proportion of patients used for categorical variables. In the efficacy analysis set, ORR was calculated with a 95% confidence interval (CI). Subgroup analyses of the effect of background factors (age, weight, genetic and chromosomal abnormality, number of prior treatments) or venetoclax dose on the safety and efficacy of venetoclax were performed using the Chi-squared or Fisher’s exact tests for categorical variables and the Wilcoxon rank-sum test for continuous variables. Results were considered statistically significant at *p* < 0.05 (two-tailed).

Statistical analyses were conducted using SAS version 9.4 (SAS Institute, Cary, NC, USA).

## Results

### Patient disposition and characteristics

**Fig. 1:**
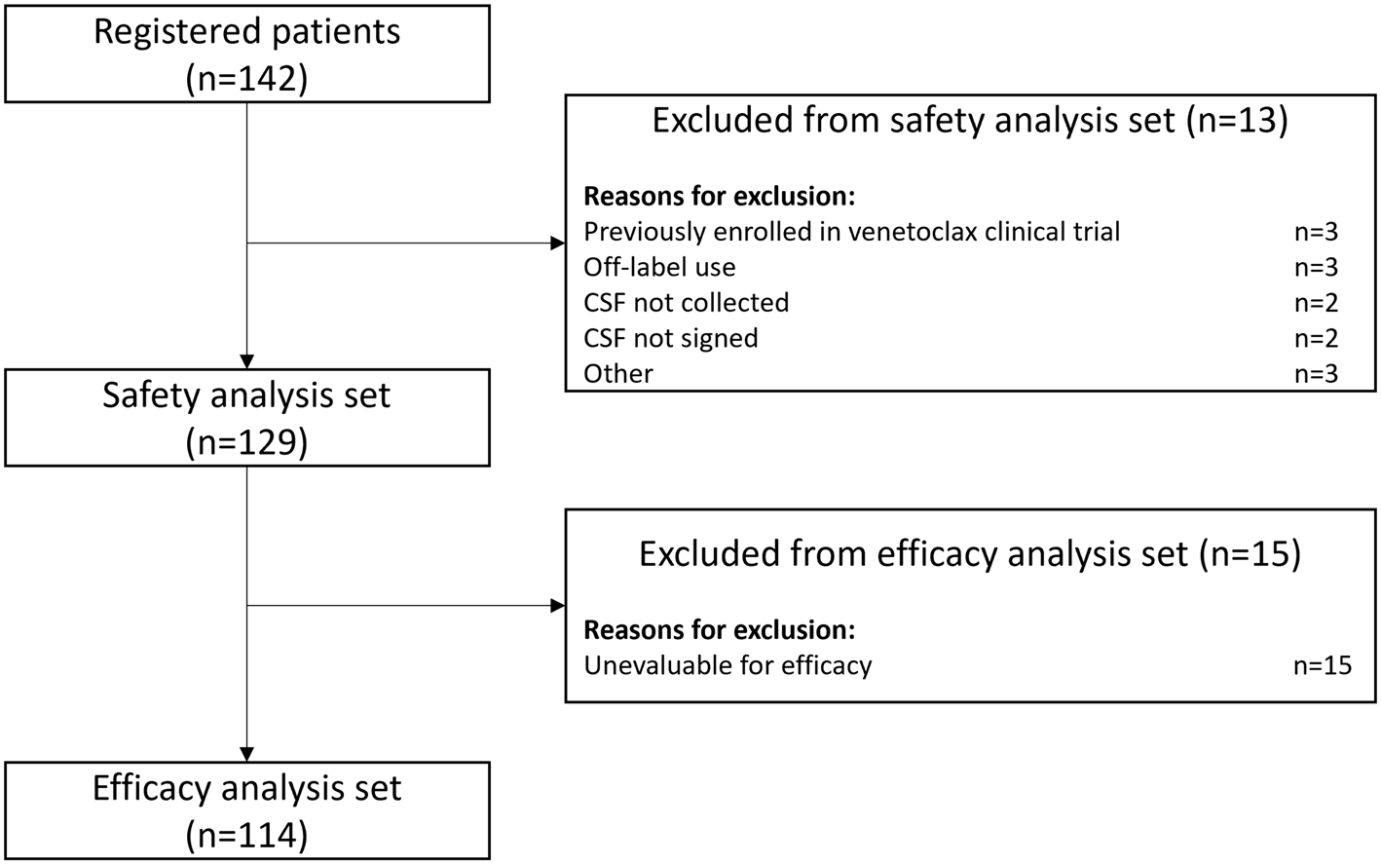
Study populations. *CRF* case report form

A total of 142 patients with R/R CLL/SLL were enrolled, of whom 129 patients were included in the safety analysis set; 13 patients were excluded because they had been previously enrolled in a venetoclax clinical trial (*n* = 3), had uncollected CRFs (*n* = 2), had been given venetoclax off-label (*n* = 3), had been transferred from the hospital (*n* = 3), or their CRF was not signed (*n* = 2). Of the 129 patients in the safety analysis set, 114 patients were included in the efficacy analysis set; 15 patients were excluded because they were unevaluable for efficacy (Fig. 1).

The majority of patients in the safety analysis set (*n* = 110) had CLL, while 19 patients were diagnosed with SLL by physicians (Table 1). Patients in the safety analysis set had a median age of 72 years (42.6% were aged ≥ 75 years), a median weight of 59.0 kg, and 72.1% were male. The median duration of CLL/SLL was 5.3 years. Patients had received a median (range) of 2 (0–10) therapies prior to venetoclax treatment, with the most common therapies being ibrutinib (*n* = 108), rituximab (*n* = 73), fludarabine (*n* = 64), and cyclophosphamide (*n* = 51). In 88 patients, ibrutinib had been received immediately prior to venetoclax treatment.

**Table 1:** Demographic and baseline clinical characteristics

	Safety analysis set *(n* = 129)
Diseases, *n* (%)	
CLL	110 (85.3)
SLL	19 (14.7)
Sex, *n* (%)	
Male	93 (72.1)
Female	36 (27.9)
Age, years	
Median (range)	72 (26–89)
Body weight, kg	*n* = 120
Median (range)	59.0 (38.6–93.0)
Presence of complications, *n* (%)	90 (69.8)
Cardiac disorders	18 (14.0)
Hepatic impairment	10 (7.8)
Renal impairment	23 (17.8)
Rai stage, *n* (%)	
0–II	60 (46.5)
III–IV	57 (44.2)
Unknown	12 (9.3)
ECOG PS, *n* (%)	
0	61 (47.3)
1	53 (41.1)
2	8 (6.2)
3–4	7 (5.4)
17p deletion, *n* (%)	
Yes	27 (20.9)
No	58 (45.0)
Unknown	44 (34.1)
*TP53* mutation, *n* (%)	
Yes	17 (13.2)
No	34 (26.4)
Unknown	78 (60.5)
*IGHV* mutation, *n* (%)	
Yes	3 (2.3)
No	12 (9.3)
Unknown	114 (88.4)
Disease duration, years	*n* = 127
Median (range)	5.3 (0.1–32.6)
Number of prior treatments	*n* = 128
Median (range)	2 (0–10)
Lymph node size, *n* (%)	
< 5 cm	74 (57.4)
≥ 5 cm	23 (17.8)
Unknown	32 (24.8)
Absolute lymphocyte count, *n* (%)	
< 25 × 10^3^ cells/μL	100 (77.5)
≥ 25 × 10^3^ cells/μL	27 (20.9)
Unknown	2 (1.6)

*CLL* Chronic lymphocytic leukemia; *ECOG PS* Eastern Cooperative Oncology Group Performance Status; *IGHV* immunoglobulin heavy chain gene; *SLL* small lymphocytic lymphoma

### Treatment

The median (range) duration of venetoclax treatment was 252.0 (3–558) days, and the median daily dose in the maintenance phase was 334.4 mg (Table 2). Sixty-five patients received a final dose of 400 mg in the ramp-up phase, 47 patients of whom reached 400 mg at 4–5 weeks after the start of treatment with venetoclax. Overall, 71 patients received venetoclax alone (55.0%) and 58 patients (45.0%) received venetoclax in combination with rituximab (Supplementary Table S1); 53 patients received rituximab in the maintenance phase, with a median (range) number of doses of 5 (1–6).

**Table 2:** Treatment details and patient disposition

	Safety analysis set (*n* = 129)
Venetoclax treatment duration, median (range), days	252.0 (3–558)
Daily dose of venetoclax (maintenance phase), mg	*n* = 91
Median (range)	334.4 (13.1–400.0)
Mean daily dose of venetoclax (maintenance phase), *n* (%)	
< 200 mg	19 (14.7)
≥ 200 to < 400 mg	29 (22.5)
400 mg	43 (33.3)
Concomitant treatment with other drugs, *n* (%)	113 (87.6)
Concomitant rituximab	58 (45.0)
Concomitant CYP3A inhibitors	14 (10.9)
Discontinuation of treatment, *n* (%)	60 (46.5)
Due to AE^a^	27 (20.9)
Due to lack of efficacy	19 (14.7)
Change of hospital	3 (2.3)
Progression of underlying disease	1 (0.8)
Other reasons	10 (7.8)

*AE* Adverse event; *CYP3A* cytochrome P450 3A

^a^Including death

In total, 60 patients (46.5%) discontinued venetoclax treatment, largely due to AEs (20.9%) or lack of effectiveness (14.7%). The most common AEs leading to discontinuation of venetoclax were neutrophil count decreased (*n* = 5), platelet count decreased (*n* = 5), and pneumonia (*n* = 4). During the ramp-up phase, 35 patients (27.1%) discontinued venetoclax due to AEs (*n* = 19) or lack of efficacy (*n* = 10).

### Efficacy

**Fig. 2:**
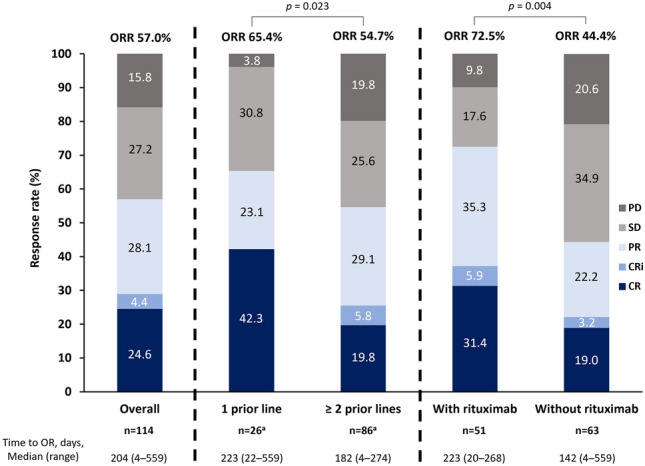
Efficacy of venetoclax in patients with relapsed/refractory chronic lymphocytic leukemia/small lymphocytic lymphoma. *CR* complete response; *CRi* complete response with incomplete bone marrow recovery; *OR* objective response; *ORR* overall response rate; *PD* progressive disease; *PR* partial response; *SD* stable disease. ^a^Total *n* = 112 in this group, which does not include 1 patient (with PD) who did not have information on prior lines of therapy recorded and 1 patient (with SD) who received venetoclax in the first line

In the efficacy analysis set (*n* = 114), ORR was 57.0% (in 65 patients), with a median (range) time to best response of 204 (4–559) days (Fig. 2), with no significant difference in ORR observed according to age, weight, or genetic and chromosomal abnormality (17p deletion, *TP53* mutation, and immunoglobulin heavy chain gene [IGHV] unmutated). Patients with a genetic or chromosomal abnormality had ORRs of at least 50%, specifically 55.3% in patients with 17p deletion, 57.7% in those with *TP53* mutation and 50.0% in those with unmutated IGHV (Supplementary Table S2).

Subgroup analyses found that ORRs were higher in patients with versus without concomitant rituximab (ORR 72.5% vs. 44.4%; *p* = 0.004), and in patients with 1 versus ≥ 2 prior lines of therapies (ORR 65.4% vs. 54.7%; *p* = 0.023; Fig. 2). Ibrutinib was the most frequently administered CLL treatment prior to venetoclax with ORR of 59.0% in 78 patients.

### Safety

Eighty-six patients (66.7%) had at least one AE, including grade ≥ 3 AEs in 55 patients (42.6%; Table 3), with no significant differences in the AE frequency observed according to age, weight, number of prior lines of treatment, or genetic and chromosomal abnormality (17p deletion, *TP53* mutation, and IGHV unmutated). The incidence of AEs was 55.0% (71/129 patients) during the ramp-up phase and 37.4% (34/91 patients) during the maintenance phase. Twenty-two patients experienced AEs after the start of rituximab during the maintenance phase.

**Table 3:** Summary of adverse events with venetoclax in patients with relapsed/refractory chronic lymphocytic leukemia/small lymphocytic lymphoma

AE, *n* (%)	Safety analysis set			
	Any AE (*n* = 129)	Grade ≥ 3 AE^b^ (*n* = 129)	AE during ramp-up phase (*n* = 129)	AE during maintenance phase (*n* = 91)
All PTs^a^	86 (66.7)	55 (42.6)	71 (55.0)	34 (37.4)
Neutrophil count decreased	29 (22.5)	26 (20.2)	22 (17.1)	12 (13.2)
White blood cell count decreased	10 (7.8)	5 (3.9)	8 (6.2)	2 (2.2)
Tumor lysis syndrome	8 (6.2)	5 (3.9)	8 (6.2)	1 (1.1)
Fever	7 (5.4)	0	5 (3.9)	2 (2.2)
Platelet count decreased	7 (5.4)	6 (4.7)	5 (3.9)	2 (2.2)
Nausea	6 (4.7)	0	3 (2.3)	3 (3.3)
Pneumonia	5 (3.9)	4 (3.1)	3 (2.3)	2 (2.2)
Anemia	4 (3.1)	3 (2.3)	4 (3.1)	0
Diarrhea	4 (3.1)	0	2 (1.6)	2 (2.2)
Hepatic function abnormal	4 (3.1)	1 (0.8)	3 (2.3)	1 (1.1)
C-reactive protein increased	4 (3.1)	0	4 (3.1)	1 (1.1)

Any grade AEs with an incidence of ≥ 3% were listed

*AE* Adverse event; *CTCAE* common terminology criteria for adverse events; *MedDRA/J* Medical Dictionary for Regulatory Activities/Japanese; *n* number of patients; *PTs* preferred terms

^a^PTs according to MedDRA/J version 25.0

^b^Graded according to the CTCAE

The most common AEs by PT of any grade were neutrophil count decreased (*n* = 29; 22.5%), white blood cell count decreased (*n* = 10; 7.8%), TLS (*n* = 8; 6.2%), platelet count decreased, and fever (*n* = 7; 5.4%).

Three of 10 patients developed fatal AEs that were causally related to venetoclax treatment, according to physician assessment (Supplementary Table S3).

#### Adverse events of special interest

All patients included in the safety analysis had received TLS preventative measures. One hundred twenty-five patients (96.9%) were monitored using blood tests, 123 patients (95.3%) had fluid loading by drinking or rehydration, and 85 patients (65.9%) received prophylactic drugs. The most common prophylactic drugs used during the ramp-up period were febuxostat (*n* = 73) and rasburicase (*n* = 10). Eight patients (6.2%) had TLS; a total of 9 TLS events were reported, including 5 of clinical TLS and 4 of laboratory TLS (Supplementary Table S4). The median (range) time from the first dose of venetoclax to the first onset of TLS was 2 (1–38) days. Six events occurred within the first 3 days of venetoclax dosing at 20 mg/day and 2 events occurred on the day of concomitant rituximab treatment. The median (range) time to recovered or recovering was 10 (4–35) days in 7 of 8 patients. Three patients discontinued venetoclax treatment due to clinical TLS and 5 patients were treated for TLS with hydration.

AEs of myelosuppression include PTs of anemia, febrile neutropenia, myelosuppression, neutropenia, pancytopenia, cytopenia, lymphocyte count decreased, neutrophil count decreased, platelet count decreased, and white blood cell count decreased. Myelosuppression occurred in 48 patients (37.2%), including grade ≥ 3 events in 37 patients (28.7%; Table 4). The median (range) time from the first dose of venetoclax to the first onset of myelosuppression was 22.0 (1–121) days, and the median (range) time to recovered or recovering was 23.5 (2–442) days in 42 of the 48 patients. Twelve patients discontinued venetoclax because of myelosuppression.

**Table 4:** Summary of myelosuppression and infection with venetoclax in patients with relapsed/refractory chronic lymphocytic leukemia/small lymphocytic lymphoma

AE, *n* (%)	Safety analysis set	
	Any AE (*n* = 129)	Grade ≥ 3 AE^e^ (*n* = 129)
Myelosuppression^a^	48 (37.2)	37 (28.7)
Neutropenia^b^	31 (24.0)	28 (21.7)
Leukopenia^c^	10 (7.8)	5 (3.9)
Thrombocytopenia^d^	8 (6.2)	7 (5.4)
Anemia	4 (3.1)	3 (2.3)
Infection	20 (15.5)	14 (10.9)
Pneumonia	5 (3.9)	4 (3.1)

Any grade myelosuppression and infection with an incidence of ≥ 3% were listed

*AE* Adverse event; *n* number of patients; *CTCAE* common terminology criteria for adverse events; *MedDRA/J* Medical Dictionary for Regulatory Activities/Japanese; *n* number of patients; *PTs* preferred terms

^a^AEs of myelosuppression include PTs according to MedDRA/J version 25.0 of anemia, febrile neutropenia, myelosuppression, neutropenia, pancytopenia, cytopenia, lymphocyte count decreased, neutrophil count decreased, platelet count decreased, and white blood cell count decreased

^b^AEs of neutropenia include PTs of neutropenia and neutrophil count decreased

^c^AEs of leukopenia include PTs of leukopenia and white blood cell count decreased

^d^AEs of thrombocytopenia include PTs of thrombocytopenia and platelet count decreased

^e^Graded according to CTCAE

Infection (defined as PTs in the system organ class ‘Infections and infestations’) occurred in 20 patients (15.5%), including grade ≥ 3 events in 14 patients (10.9%; Table 4). The median (range) time from the first dose of venetoclax to the first onset of infection was 30.5 (1–257) days, and the median (range) time to recovered or recovering was 15.0 (3–63) days in 14 of the 20 patients. Eleven patients discontinued venetoclax because of infection.

## Discussion

This study is the first large-scale, real-world efficacy and safety evaluation of venetoclax in R/R CLL/SLL patients in Japan. Venetoclax had a manageable safety profile and was efficacious.

The ORR was 57.0%, with a significantly higher response in patients receiving concomitant rituximab than in those receiving venetoclax alone (ORR 72.5% vs. 44.4%; *p* = 0.004). In the MURANO study, the ORR was 93.3% with venetoclax plus rituximab [6]. In contrast, in the M13-834 study, the ORR was 67.0% with venetoclax plus rituximab and 100.0% with venetoclax [7]. The ORR in the current study was lower than the ORR from real-world studies in other countries, for example, 66.0–85.0% in Italy [9] and 95.3% in Poland [10], which could be due to differences in patient populations, study methods or the use of concomitant rituximab. In the Polish report, in which the ORR was 95.3%, all the patients treated with venetoclax also received rituximab [10]. Similarly, real-world Italian studies reported the highest ORR (85.0%) in patients who received concomitant rituximab, whereas the ORR in patients not receiving rituximab was 66% or 75% [9], which is closer to the result of this study.

Although it is stated in the Japanese package insert that "rituximab should be administered concomitantly from the start of the maintenance period unless administration of rituximab is difficult", 45% of patients in this study received rituximab. The group of patients not receiving rituximab were not markedly different from those receiving rituximab, except that the rituximab group included a lower proportion of patients with Rai stage 0–II (53.7% vs. 43.7%), and Eastern Cooperative Oncology Group performance status of 0 (56.9% vs. 39.4%). It is considered that rituximab was not concomitantly used in patients in relatively poor condition. However, the reason why rituximab was not received was not collected directly, and therefore, it is difficult to speculate on the reasons for the low use of rituximab in this study.

The overall incidence of AEs was 66.7% in this study. The incidence of AEs was 100.0% in the M13-834 study [7], and 99.2% in the MURANO study [6]. The lower incidence of AEs in the current study is likely due to the limitations of AE monitoring in real-world clinical practice compared with rigorous monitoring and follow-up in clinical trials, and also due to differences in assessment of AEs between these two different study types.

Although comparisons across trials should be made with caution due to differences in study populations and trial methods, overall, the results of this study support the efficacy and safety findings of previous studies of venetoclax in patients with R/R CLL/SLL.

TLS occurs when tumor cells release their contents into the bloodstream spontaneously or in response to therapy [11]. In this study, TLS occurred in 6.2% of patients, similar to that reported for studies in the clinical practice setting in other countries (1.2–16.0%) [6, 12–16]. Previous studies have indicated that the risk of TLS is higher when initiating venetoclax treatment and during dose ramp-up [17]. Consistent with this observation, all but one TLS event in the current study occurred during the venetoclax dose ramp-up phase. Thus, caution is advised during this phase; the TLS risk should be managed with prophylactic strategies, such as aggressive hydration, management of hyperuricemia with anti-uricemics (e.g., allopurinol, febuxostat, and rasburicase), and frequent electrolyte monitoring [18, 19].

Neutropenia (include PTs of neutropenia and neutrophil count decreased) was the most common AE in this study, while the most common AE in the M13-834 [7] and MURANO [6] studies was neutropenia (92.0% and 60.8%, respectively). The AEs observed in the current study were consistent with the known safety profile of venetoclax. The incidence of AEs was higher during the ramp-up phase than during the maintenance phase, and the combination with rituximab is unlikely to affect the occurrence of AEs.

Myelosuppression and infections were the most common AEs associated with treatment discontinuation. Such events are common AEs with other agents used in the treatment of CLL and infection risk is increased by the immunosuppressive effects of CLL itself [20]. Patients should be carefully evaluated for their risk of myelosuppression and infection before starting treatment, taking into account their disease status, vaccination status, comorbidities, history of infections (e.g. tuberculosis, herpes zoster), baseline blood cell counts, and prior and current treatments. Once evaluated, individualized decisions can be made about the use of prophylactic growth factors and/or anti-infective agents. Most myelosuppression and infection events in this study resolved without sequelae and were manageable.

This study had a few limitations. Firstly, there was no comparator group, meaning that the results cannot be compared with other approved treatments. Secondly, all results depended on investigator reporting, which may have led to reporter bias. Lastly, the study was conducted only in Japan, limiting the generalizability of the results in terms of racial diversity. However, a strength of this study is the real-world setting and resulting heterogenous patient population, which provides advantages for assessing drug effects when compared to the very homogenous patient populations included in clinical trials.

## Conclusions

This study confirmed the efficacy and safety of venetoclax in Japan in a real-world clinical setting in patients with R/R CLL/SLL. No notable differences in efficacy were observed from previous studies of venetoclax, and no new safety issues were identified.

## Supplementary Information

Below is the link to the electronic supplementary material.

Supplementary file1 (DOCX 29 KB)

## Data Availability

The datasets used and/or analyzed during the current study are available from the corresponding author on reasonable request.
