# Correction: Venetoclax treatment for chronic lymphocytic leukemia/small lymphocytic leukemia in Japan: post-marketing surveillance

**DOI:** 10.1007/s12185-025-03978-2

**Published:** 2025-04-11

**Authors:** Tomoki Ito, Tomohiko Kamimura, Toru Kiguchi, Koji Kato, Risa Takenaka, Mariko Kobayashi, Ayumi Ito, Mizu Sakai, Koji Izutsu

**Affiliations:** 1 Department of Hematology, Kansai Medical University Hospital, 2-3-1 Shinmachi, Hirakata City, Osaka Japan; 2 Department of Hematology, Harasanshin Hospital, Fukuoka, Japan; 3 Department of Diabetes, Endocrinology and Hematology, Dokkyo Medical University Saitama Medical Center, Saitama, Japan; 4 Department of Hematology, Oncology and Cardiovascular Medicine, Kyushu University Hospital, Fukuoka, Japan; 5 AbbVie GK, Tokyo, Japan; 6 Department of Hematology, National Cancer Center Hospital, Tokyo, Japan

**Correction: International Journal of Hematology (2024) 120:613–620** 10.1007/s12185-024-03832-x

In this article, the sentence beginning ‘Patients with a genetic or chromosomal abnormality had ORRs…’ in the Efficacy section was incorrectly given as ‘Patients with a genetic or chromosomal abnormality had ORRs of at least 50%, specifically 55.3% in patients with 17p deletion, 57.7% in those with TP53 mutation and 50.0% in those with unmutated IGHV (Supplementary Table S2).’ and should have read ‘Patients with a genetic or chromosomal abnormality had ORRs of at least 50%, specifically 61.5% in patients with 17p deletion, 56.3% in those with TP53 mutation and 50.0% in those with unmutated IGHV (Supplementary Table S2).’.

