# Supplementary material for: Venetoclax treatment for chronic lymphocytic leukemia/small lymphocytic leukemia in Japan: post-marketing surveillance
Source: Int J Hematol. 2024 Aug 21;120(5):613–20. doi: 10.1007/s12185-024-03832-x (PMC11513738; doi:10.1007/s12185-024-03832-x)
Supplement: Supplementary file 1 — Supplementary file1 (DOCX 29 KB) [file 12185_2024_3832_MOESM1_ESM.docx]

# Supplemental data

# Supplementary Table S1.

Demographic and baseline clinical characteristics in patients who received and those who did not receive concomitant rituximab.

|  | **Safety analysis set (n=129)** | |
| --- | --- | --- |
|  | **With rituximab (n=58)** | **Without rituximab (n=71)** |
| Sex, n (%) |  |  |
| Male | 41 (70.7) | 52 (73.2) |
| Female | 17 (29.3) | 19 (26.8) |
| Age, years |  |  |
| Median (range) | 71.5 (51–85) | 73 (26-89) |
| Body weight, kg |  |  |
| Median (range) | 57.75 (40.3–93.0) | 60.2 (38.6-82.0) |
| Presence of complications, n (%) | 42 (72.4) | 49 (69.0) |
| Cardiac disorders | 6 (10.3) | 12 (16.9) |
| Hepatic impairment | 6 (10.3) | 4 (5.6) |
| Renal impairment | 9 (15.5) | 14 (19.7) |
| Rai stage, n (%) |  |  |
| 0–II | 29 (53.7) | 31 (43.7) |
| III–IV | 23 (42.6) | 34 (47.9) |
| Unknown | 6 (10.3) | 6 (8.5) |
| ECOG PS, n (%) |  |  |
| 0 | 33 (56.9) | 28 (39.4) |
| 1 | 19 (32.8) | 34 (47.9) |
| 2 | 3 (5.2) | 5 (7.0) |
| 3–4 | 3 (5.2) | 4 (5.6) |
| 17p deletion, n (%) |  |  |
| Yes | 16 (27.6) | 12 (16.9) |
| No | 24 (41.4) | 33 (46.5) |
| Unknown | 18 (31.0) | 26 (36.6) |
| *TP53* mutation, n (%) |  |  |
| Yes | 9 (15.5) | 8 (11.3) |
| No | 14 (24.1) | 20 (28.2) |
| Unknown | 35 (60.3) | 43 (60.6) |
| *IGHV* mutation, n (%) |  |  |
| Yes | 2 (3.4) | 1 (1.4) |
| No | 4 (6.9) | 8 (11.3) |
| Unknown | 52 (89.7) | 62 (87.3) |
| Disease duration, years | n=57 | n=70 |
| Median (range) | 5.5 (0.3–32.6) | 5.2 (0.1-22.4) |
| Number of prior treatments |  |  |
| Median (range) | 2 (1–10) | 3 (0-7) |

CLL, chronic lymphocytic leukemia; ECOG PS, Eastern Cooperative Oncology Group performance status; *IGHV*, immunoglobulin heavy chain gene; SLL, small lymphocytic lymphoma.

## Supplementary Table S2.

Efficacy of venetoclax in patients with genetic and/or chromosomal abnormalities.

|  | **17p deletion (n=26)** | ***TP53* mutation (n=16)^a^** | ***IGHV* unmutated (n=10)** |
| --- | --- | --- | --- |
| ORR, n (%) | 16 (61.5) | 9 (56.3) | 5 (50.0) |
| CR | 9 (34.6) | 5 (31.3) | 3 (30.0) |
| CRi | 0 | 1 (6.3) | 0 |
| PR | 7 (26.9) | 3 (18.8) | 2 (20.0) |
| nPR | 0 | 0 | 0 |
| SD | 5 (19.2) | 4 (25.0) | 5 (50.0) |
| PD | 5 (19.2) | 3 (18.8) | 0 |
| Time to OR, days |  |  |  |
| Median (range) | 219.5 (20–271) | 239 (20–271) | 92 (13–249) |

^a^In this group, 8 patients also had a 17p deletion (CR: n=2; PR: n=3; SD: n=1; PD: n=2), 5 patients did not have a 17p deletion (CR: n=1; SD: n=3; PD: n=1), and 3 patients did not have information on other genetic or chromosomal abnormalities.

CR, complete response; CRi, complete response with incomplete bone marrow recovery; *IGHV*, immunoglobulin heavy chain gene; nPR, nodular partial response, OR, objective response; ORR, overall response rate; PD, progressive disease; PR, partial response; SD, stable disease.

## Supplementary Table S3.

Summary of patients who had developed fatal adverse events causally related to venetoclax in whom the outcome was death.

| **Parameter** | | **Age, decade, years** | **Sex** | **Primary disease** | **ECOG PS** | **Rai stage** | **Treatment history^a^** | **Rituximab use** | **Clinical history** | **ADR(s)** | **Peripheral blood lymphocyte count (/μL)** | **Enlarged lymph node in the long axis (cm)** |
| --- | --- | --- | --- | --- | --- | --- | --- | --- | --- | --- | --- | --- |
|  | **Case number 1** | 70s | M | CLL | 1 | IV | 4  fludarabine, rituximab, bendamustine, ibrutinib | No | Early gastric cancer, bacterial pneumonia | Pneumococcal pneumonia; sepsis | 28,517 | 20 |
|  |  | After confirming that the bacterial pneumonia had subsided, venetoclax was initiated as 5th line therapy in July 2020 at a dose of 20 mg. The dose was then increased to 50 mg one week later. On Day 13, the patient suddenly developed a fever, and an X-ray did not reveal any signs of pneumonia. Levofloxacin was administered, but he experienced vomiting, bradycardia, and cardiac arrest that night, and unfortunately died the following day. *Pneumococcal pneumoniae* was detected in the patient's blood culture, leading to the conclusion that septic shock caused by bacterial pneumonia was the cause of death. | | | | | | | | | | |
|  | **Case number 2** | 60s | M | SLL | 0 | IV | 3  rituximab, bendamustine, cyclophosphamide, ibrutinib | No | CKD | Staphylococcal septicemia | 2,992 | 2 |
|  |  | Venetoclax was started at 20 mg as the 4th line therapy in January 2020. Due to grade4 neutropenia and grade3 cytopenia, the dose of venetoclax was increased to 100 mg after approximately 1 and a half months with dose interruption and dose reduction. The patient continued to experience grade 4 neutropenia and grade3 cytopenia and was switched to venetoclax 50 mg every other day, but was discontinued in May due to problem with shunt during hemodialysis for CKD. PTA was performed, but 2 days later, he developed staphylococcal sepsis and died in June. | | | | | | | | | | |
|  | **Case number 3** | 70s | F | CLL | 0 | III | 4  fludarabine, rituximab, cyclophosphamide, ibrutinib | No | Hyperuricemia, reflux esophagitis, type 2 diabetes mellitus,  peripheral neuropathy,  steroid osteoporosis | Decreased platelet count; pulmonary hemorrhage | 29,107 | 5.3 |
|  |  | Venetoclax was initiated at a dose of 20 mg as 5th line therapy in August 2020. The patient developed a fever on day 2 and pseudomembranous enteritis on day 3, but the chest X-ray was normal. On day 4, she experienced pulmonary hemorrhage and respiratory failure the following day, with chest CT revealing multiple patchy shadows in the bilateral lung fields. Platelet count decreased from 52,000 to 21,000 /μL. The patient died on the sixth day due to progression of respiratory failure, which was judged to be due to progression of CLL based on the rapid deterioration of lymphocyte counts. | | | | | | | | | | |

^a^CLL/SLL treatment line number

ADR, adverse drug reaction; CKD, chronic kidney disease; CLL, chronic lymphocytic leukemia; CT, computed tomography; ECOG PS, Eastern Cooperative Oncology Group performance status; F, female; M, male; PTA, percutaneous transluminal angioplasty; SLL, small lymphocytic lymphoma.

## Supplementary Table S4.

Summary of patients who had developed tumor lysis syndrome during treatment with venetoclax.

| **Parameter** | | Age, decade, years | Sex | Abnormal renal function | Lymph node size, cm | Absolute lymphocyte count, /μL | Receiving prophylactic hydration | Receiving prophylactic medication | Clinical or laboratory TLS | Time to detection of TLS, days^a^ | Outcome | Venetoclax discontinued |
| --- | --- | --- | --- | --- | --- | --- | --- | --- | --- | --- | --- | --- |
| **Case number** | **1** | 60s | M | No | 1 | 516 | Yes | Yes | CTLS | 16 | Recovering | Yes |
|  | **2** | 70s | M | No | 0 | 918 | Yes | Yes | CTLS | 3 | Not recovered | Yes |
|  | **3** | 80s | M | Yes | 6 | 5,812.5 | Yes | Yes | CTLS | 1 | Recovered | No |
|  | **4** | 70s | F | No | 1.3 | 30,861 | Yes | Yes | CTLS | 38 | Recovering | Yes |
|  | **5** | 70s | M | No | 2 | 15,576.3 | Yes | Yes | LTLS; CTLS | 2; 170 | Recovering; recovering | No |
|  | **6** | 80s | M | No | NA | 140.7 | Yes | Yes | LTLS | 2 | Recovered | No |
|  | **7** | 80s | M | No | 3 | 22,800 | Yes | Yes | LTLS | 1 | Recovering | No |
|  | **8** | 70s | F | No | 4.2 | 91,000 | Yes | No | LTLS | 1 | Recovered | No |

^a^From start of venetoclax administration to onset of symptoms.

NA, data not available; CTLS, clinical TLS; F, female; LTLS, laboratory TLS; M, male; TLS, tumor lysis syndrome.
